# Influence of High Pass Filter Settings on Motor Evoked Potentials

**DOI:** 10.3389/fnins.2021.665258

**Published:** 2021-04-23

**Authors:** Petyo Nikolov, Shady S. Hassan, Alfons Schnitzler, Stefan J. Groiss

**Affiliations:** ^1^Institute of Clinical Neuroscience and Medical Psychology, Medical Faculty, Heinrich Heine University Düsseldorf, Düsseldorf, Germany; ^2^Department of Neurology, Medical Faculty, Heinrich Heine University Düsseldorf, Düsseldorf, Germany; ^3^Department of Neurology, Medical Faculty, Assiut University Hospital, Assiut, Egypt

**Keywords:** transcramial magnetic stimulation, motor evoked potential, high pass filter, notch filter, artifacts

## Abstract

**Objective:**

Motor evoked potentials (MEP), obtained by transcranial magnetic stimulation (TMS) are a common tool in clinical research and diagnostic. Nevertheless, reports regarding the influence of filter settings on MEP are sparse. Here, we compared MEP amplitudes and signal to noise ratio (SNR) using multiple high pass filter (HPF) and notch filter settings.

**Materials and Methods:**

Twenty healthy subjects were enrolled in the study. Recruitment curves were obtained with HPF settings varied at 10, 20, 50, and 100 Hz. The four HPF settings were tested both with and without 50 Hz active notch filter. Low pass filter was kept constant at 5 kHz.

**Results:**

MEP amplitudes with HPF at 10 and 20 Hz were significantly higher than at 100 Hz, regardless of the notch filter. However, SNR did not differ among HPF settings. An active notch filter significantly improved SNR.

**Conclusion:**

The reduction in MEP amplitudes with HPF above 20 Hz may be due to noise reduction, since the different HPF conditions did not alter SNR. Thus, higher HPF above 50 Hz may be an option to reduce noise, the use of a notch filter may even improve SNR.

**Significance:**

Our findings are relevant for the selection of filter settings and might be of importance to any researcher who utilizes TMS-MEP.

## Introduction

Transcranial magnetic stimulation (TMS) holds clinical, diagnostic and therapeutic value, with a growing number of established TMS protocols. Implying TMS, motor cortex excitability is often studied on the muscle level via motor evoked potentials (MEP). Here, the frequency of the recordings must be appropriately filtered to avoid any interference with background noise. Usually, before starting the stimulation session, the researcher needs to adjust the high pass filter (HPF), as well as the low pass filter (LPF) setting. In this way, only signal frequencies that lay between the two filter settings are being recorded.

Besides HPF and LPF, also band-pass and band-stop filters (also known as notch filters) are commonly used in a typical TMS-MEP session. The most common notch filter in Europe is set at 50 Hz because of the ripple interference artifact of the alternating current used on the continent.

In this context, there is still little information, which filter setting is the best for performing TMS-MEP recordings. The settings, used for HPF and LPF often differ in the literature (Nakamura et al., 1997; Daskalakis et al., 2004; Di Lazzaro et al., 2006; Hanajima et al., 2009) and there is not a clear recommendation if the notch filter should be kept active. Here, we explored the quality of TMS-MEP recordings with different HPF and notch filter settings, by comparing MEP amplitudes between them. We focused only on the HPF and kept the LPF constant.

## Materials and Methods

### Participants

The current study took place at the Department of Neurology in the Düsseldorf University Hospital. In it, we included twenty healthy subjects (13 male; 7 female, mean age = 29 ± 1.6 SEM years) who participated in the study after giving their explicit written consent. The study was performed in accordance with the Helsinki Declaration (World Medical Association, 2013) and was approved by the Ethics Committee of the Medical Faculty at the Heinrich Heine University Düsseldorf.

Exclusion criteria were contraindication to TMS (e.g., due to metallic and/or magnetic implants), severe intestinal, neurological, or psychiatric diseases, the use of any medication acting on the central nervous system (e.g., benzodiazepines, anti-epileptic, and/or psychotropic drugs), blood clotting dysfunction, pregnancy, and diagnosed peripheral/retinal neuropathy.

### Transcranial Magnetic Stimulation

Transcranial magnetic stimulation was applied by Magstim^TM^ magnetic stimulator (The Magstim Co. Ltd, Whitland, United Kingdom) through a figure-of-eight coil. Single-pulse stimulation was used throughout the whole study. The coil was placed above the left primary motor cortex (M1) tangentially to the scalp, with the coil handle pointing backwards and laterally at a 45° angle to the sagittal plane to ensure a posterior-anterior current direction in the brain (Rothwell, 1997). This configuration aims to trans-synaptically activate the corticospinal system by means of horizontal cortico-cortical connections (Di Lazzaro et al., 2004). After determination of the individual TMS hotspot, resting motor threshold (RMT), as well as active motor threshold (AMT) were obtained for each participant. RMT was defined as the lowest stimulation intensity that evoked 100 μV response during complete relaxation of the right first dorsal interosseous muscle (FDI) in at least 5 of 10 trials using the relative frequency method (Rossini et al., 2015). Analogously, AMT was defined as the lowest intensity that evoked consistent 100 μV FDI response during 5–10% of maximal muscle contraction. Throughout the article, intensity is measured as percent from the maximal stimulator output.

### Electromyographic Recording

EMG signals were recorded from the right FDI muscle by means of disposable Ag-AgCl surface electrodes (20 × 15 mm, Ambu^TM^ Neuroline 700, Penang, Malaysia). The active electrode was placed on the muscle belly, whereas the inactive electrode was located over the base of the metacarpophalangeal joint of the index finger. EMG signals were amplified (Digitimer D360, Digitimer Ltd, Hertfordshire, United Kingdom), filtered and stored on a desktop computer for off-line analysis. Here, we tested four different HPF settings: 10, 20, 50, and 100 Hz. Each of the HPF settings was tested both with and without an active 50 Hz notch filter, which resulted in eight filter settings per subject. The LPF was constantly kept at 5 kHz throughout all experiments.

### Experimental Design

Participants were seated in a comfortable reclining chair with arms placed on cushioned armrests during the entire experiment. Subsequently, electrodes were attached to the right FDI in belly-tendon montage. Then, the individual TMS-hotspot was determined in steps of 0.5 to 1 cm, starting 5cm lateral and 1.5 cm anterior of the vertex, as the stimulation site where suprathreshold TMS produced the largest MEP-FDI amplitude. The hotspot was marked directly on the scalp with a soft-tip pen to insure constant placement of the TMS coil. Based on this hotspot, TMS intensities for RMT and AMT were determined multiple times for each of the eight filter settings (10, 20, 50, and 100 Hz: once with active notch filter, and once without).

After determining RMT and AMT, recruitment curves were obtained at each of the eight filter settings. Recruitment curves were generated by single-pulse TMS, based on 100, 110, 120, 130, and 140% RMT intensity. Each intensity configuration consisted of 12 trials, resulting in 60 trials per filter setting. The order of the intensity configurations was randomized across and within subjects. Through the course of the entire experiment, muscle relaxation was monitored by an oscilloscope (Rigol DS1074B, Hirschau, Germany). Subjects were instructed to look at a fixation cross centered in front of them and silently count the number of magnetic pulses applied to maintain similar level of attention.

### Data Analysis and Statistical Evaluation

EMG data analysis was performed with Signal Software (Cambridge Electronic Design, Cambridge, United Kingdom). Trials were visually inspected. Trials showing voluntary EMG activity immediately before the TMS pulse, as well as trials where no TMS pulse was present due to technical reasons, were rejected from the analysis. On average, 16 ± 4 SEM from a total of 480 trials were rejected per subject. Maximum peak-to-peak MEP amplitudes and maximum noise amplitudes during the time period of 20 ms before the TMS pulse, were determined for each trial. Subsequently, peak-to-peak amplitudes were averaged over all trials of each filter setting to gain MEP-amplitudes and noise amplitudes. SNR was calculated as a ratio of MEP amplitudes/noise amplitudes.

Statistical evaluation was performed with SPSS and R. Shapiro-Wilk test was used to test for normality. Generalized estimating equation (GEE) model was used to compare intensity at RMT and AMT, respectively, between filter settings. Here, threshold intensity was the dependent variable, while HPF setting (10, 20, 50, and 100 Hz) and notch filter setting (active; non-active) were co-factors.

MEP amplitudes, noise amplitudes and SNR, obtained from the recruitment curves were evaluated also with GEE. In the model, signal amplitudes and SNR were the dependent variables. HPF setting (10, 20, 50, and 100 Hz), notch filter setting (active, non-active) and stimulation intensity (100, 110, 120, 130, and 140% RMT) were co-factors.

## Results

### RMT and AMT

Intensity at RMT was not significantly affected by HPF setting (Wald-Chi^2^ = 0.25; *p* = 0.96; mean = 44.6 ± 1% SEM for 10 Hz; 45 ± 1.3% SEM for 20 Hz; 45.4 ± 1.4% SEM for 50 Hz, and 45.5 ± 1.5% SEM for 100 Hz). It was also not significantly affected by the notch filter setting (Wald-Chi^2^ = 0.013; *p* = 0.9; mean = 45 ± 1% SEM with active notch filter; 45.2 ± 1% SEM with non-active notch filter).

Analogously, intensity at AMT was not significantly affected either by HPF setting (Wald-Chi^2^ = 0.63; *p* = 0.88; mean = 33.2 ± 1.2% SEM for 10 Hz; 33.8 ± 1.2% SEM for 20 Hz; 34.4 ± 1.2% SEM for 50 Hz; 34.4 ± 1.2% SEM for 100 Hz), or notch filter (Wald-Chi^2^ = 0.023; *p* = 0.87; mean = 34.1 ± 0.8% SEM with active notch filter; 33.9 ± 0.9% SEM with non-active notch filter). For an overview of RMT and AMT mean values please see Table 1.

**TABLE 1:** Summary of mean resting motor threshold (RMT) and active motor threshold (AMT) values between filter settings, given as percentage from maximal stimulator output ± SEM.

RMT		AMT	
10 Hz	44.6 ± 1%	10 Hz	33.2 ± 1.2%
20 Hz	45 ± 1.3%	20 Hz	33.8 ± 1.2%
50 Hz	45.4 ± 1.4%	50 Hz	34.4 ± 1.2%
100 Hz	45.5 ± 1.5%	100 Hz	34.4 ± 1.2%

### MEP Amplitudes

When comparing MEP-amplitudes, obtained in form of recruitment curves, notch filter was significant factor (*z* = −2.54, *p* = 0.011). Mean MEP-amplitudes with active notch filter (mean = 1.085 ± 0.057 mV SEM) were smaller than mean MEP-amplitudes without notch filter (mean = 1.253 ± 0.066 mV SEM). Further, HPF with 100 Hz was a significant factor (*z* = −4.75, *p* < 0.001), resulting in smaller MEP-amplitudes (mean MEP-amplitude 100 Hz = 0.89 ± 0.06 mV SEM). On the other hand, 50, 20, and 10 Hz HPF were non-significant factors (*p* > 0.05 for all three; mean MEP-amplitude 50 Hz = 1.17 ± 0.08 mV SEM; 20 Hz = 1.3 ± 0.09 mV SEM; 10 Hz = 1.3 ± 0.9 mV SEM). As expected, stimulation intensity significantly affected MEP-amplitudes during recruitment curves (*p* < 0.001), with MEP-amplitudes significantly higher for each intensity increment: mean 100%RMT = 0.18 ± 0.03 mV SEM; 110%RMT = 0.45 ± 0.05 mV SEM; 120%RMT = 0.84 ± 0.08 mV SEM; 130%RMT = 1.48 ± 0.11 SEM; 140%RMT = 1.89 ± 0.14 mV SEM.

### Noise Amplitudes and Signal-Noise Ratio

Considering noise amplitudes, notch filter was significant factor (*z* = −21.7; *p* < 0.001), which resulted in significant smaller noise amplitudes (mean noise amplitude with notch filter = 0.02 ± 0.0003 mV; without notch filter = 0.07 ± 0.0024 mV). Further, 100 Hz HPF (*z* = −10.08; *p* < 0.001) and 50 Hz HPF (*z* = −2.55; *p* = 0.01) were significant factors, resulting in smaller noise amplitudes (mean noise amplitude 100 Hz = 0.033 ± 0.001 mV SEM; 50 Hz = 0.055 ± 0.002 mV SEM). In contrast, 10 Hz and 20 Hz HPF were non-significant (mean noise amplitude with 20 Hz = 0.057 ± 0.003 mV SEM; 10 Hz = 0.064 ± 0.003 mV SEM *p* > 0.05). Also, stimulation intensity did not significantly affect noise amplitudes (*p* > 0.05 for all stimulation increments).

Regarding SNR, notch filter was significant factor (*z* = 8.2; *p* < 0.001), which increased SNR significantly (mean SNR with notch filter = 42.64 ± 2.4 SEM; without notch filter = 23.67 ± 1.51 SEM). On the other hand, SNR was not significantly affected by the HPF settings (*p* > 0.05 for all HPF settings). As in the case with the recruitment curve of MEP-amplitudes, SNR increased significantly with each TMS intensity increment: mean SNR 100%RMT = 4.38 ± 2.41 SEM; 110%RMT = 13.96 ± 1.2 SEM; 120%RMT = 30.16 ± 2.25 SEM; 130%RMT = 52.01 ± 3.63 SEM; 140%RMT = 65.1 ± 4.12 SEM.

## Discussion and Conclusion

To our knowledge, this is the first study, systematically exploring different high pass filter setting for TMS-MEP recordings. It has two main findings. First, SNR is not affected by HPF settings up to 100 Hz, as both MEP- and noise recordings show smaller amplitudes with increasing HPF. Second, a notch filter of 50 Hz significantly improves SNR by reducing noise.

As the active frequency of human muscle fibers lies around 40 Hz (McAuley et al., 1997), it is largely believed that the HPF should not be lifted above 10 Hz to avoid filtering out muscle responses along with the background noise. Hence, HPF above 10 Hz is rarely applied in TMS-MEP protocols. In this relation, past reports suggest that HPF > 20 Hz, could suppress the meaningful muscle signal (Zschorlich, 1989). This notion is backed by more recent report, which showed the optimal HPF bandwidth for the facial musculature to be between 15 and 25 Hz (van Boxtel, 2001). In accordance, most of the present TMS protocols use HPF in the range of 10–25 Hz.

**FIGURE 1:**
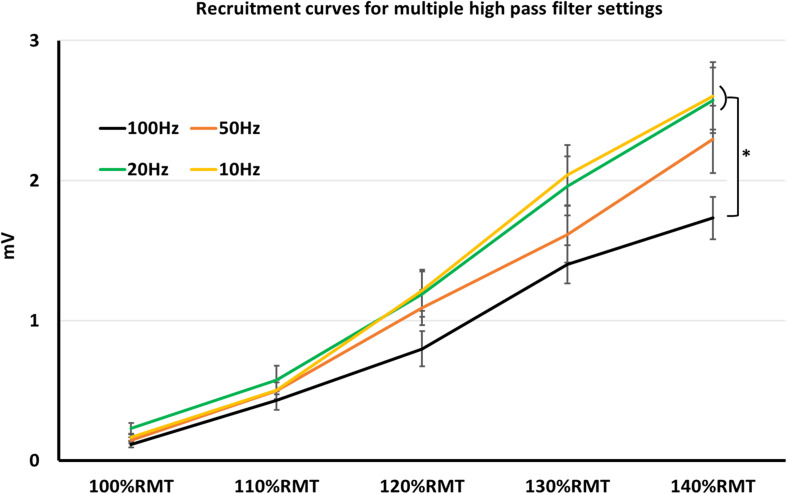
Recruitment curves, obtained in multiple high pass filter settings. Here, MEP-values with high pass filter at 10–20 Hz were significantly higher than at 100 Hz. *p < 0.05; indices represent SEM.

**FIGURE 2:**
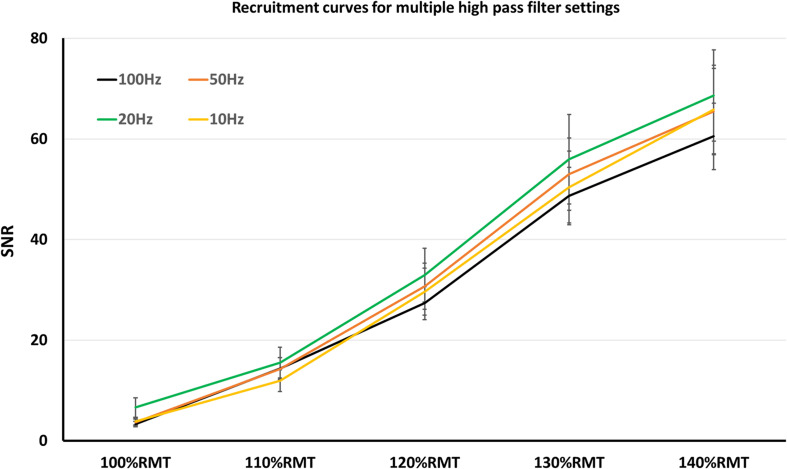
Recruitment curves, obtained in multiple high pass filter settings. Here, SNR did not significantly differ among the high pass filter settings.

Nevertheless, lifting the HPF up is a conceivable strategy to avoid artifacts. One of the most common artifacts when recording surface MEP signals is the baseline fluctuation noise. Electrode-skin discharges, as well as electrical drifts, due to the technical equipment, can cause such noises. A HPF setting > 20 Hz could successfully suppress most of the baseline fluctuation noise, however, at the expense of the real signal (Stålberg et al., 1986). Indeed, our results showed significantly smaller MEP-amplitudes with 100 Hz HPF, which argues for signal reduction when using HPF > 20 Hz. However, the signal reduction occurs equally in noise and MEP recordings, thus leaving the SNR unaffected (Figure 1). Furthermore, the signal reduction occurs only when supra-threshold stimuli (producing > 1 mV MEP amplitude) are used. With lower simulation intensity, differences in MEP signals between HPF settings seem to diminish (Figure 2).

Another common artifact in TMS-MEP protocols is the 50 Hz ripple noise, which is caused by the alternating current used in Europe. A reliable way to avoid the 50 Hz ripple artifact is to place an active notch filter during recordings. To our knowledge, the current study is the first one to systematically evaluate the impact of 50 Hz active notch filter on TMS recordings. Here, noise was reduced with more than 300%, and SNR was improved by almost 100% when using active notch filter. Hence, we conclude that the notch may be useful to improve SNR and its implementation in TMS protocols be considered.

It is worth mentioning that the impact of HPF setting may slightly vary depending on the specific environmental and study setup of each laboratory. Ideally, different HPF settings for each study setting may be investigated by each laboratory to ensure to choose the optimal one.

Taken together, HPF up to 100 Hz may be feasible when measuring TMS-MEP, as it does not reduce SNR. On the other hand, the use of a notch filter may be considered, because it reduces noise and improves SNR.

## Data Availability Statement

The raw data supporting the conclusions of this article will be made available by the authors, without undue reservation.

## Ethics Statement

The studies involving human participants were reviewed and approved by Ethics Committee, Heinrich Heine University Düsseldorf. The patients/participants provided their written informed consent to participate in this study.

## Author Contributions

PN organized and executed the research project, executed the statistical analysis, and wrote the first draft of manuscript preparation. SH conceived the research project, designed the statistical analysis, and prepared the review and critique of the manuscript. AS organized the research project, reviewed the statistical analysis, and prepared the review and critique of the manuscript. SG conceived and organized the research project, conceived the executed the statistical analysis, and prepared the review and critique of the manuscript. All authors contributed to the article and approved the submitted version.

## Conflict of Interest

The authors declare that the research was conducted in the absence of any commercial or financial relationships that could be construed as a potential conflict of interest.

## Abbreviations

AMT: Active Motor Threshold
EMG: Electromyography
FDI: First Dorsal Interosseous
GEE: General Estimating Equations
HPF: High Pass Filter
LPF: Low Pass Filter
MEP: Motor Evoked Potentials
RMT: Resting Motor Threshold
SEM: Standard Error of the Mean
SNR: Signal to Noise Ratio
TMS: Transcranial Magnetic Stimulation.
